# Supplementary material for: Zero-shot learning of aerosol optical properties with graph neural networks
Source: Sci Rep. 2023 Oct 31;13:18777. doi: 10.1038/s41598-023-45235-8 (PMC10618469; doi:10.1038/s41598-023-45235-8)
Supplement: Supplementary file 1 — Supplementary Information. [file 41598_2023_45235_MOESM1_ESM.pdf]

Supplementary Information: Zero-Shot Learning of Aerosol Optical Properties with Graph Neural Networks

K.D. Lamb[a] and P. Gentine[a]

[a]Department of Earth and Environmental Engineering, Columbia University

In this Supplementary Information we provide a more detailed description of the electromagnetic scattering problem (Section S1) and the graph neural network modeling approaches that we tested for the prediction of aerosol optical properties (Section S2). We discuss only approaches relevant to the current work here; additional information about graph neural networks and their applications can be found in several recent comprehensive review papers [1, 2, 3, 4, 5].

S1 Electromagnetic scattering from multiple spheres

Mie Theory

Electromagnetic scattering from a sphere is known as the Lorenz-Mie solution to Maxwell's equations [6, 7]. Assuming a plane wave is incident on the sphere, the solution can be found by decomposing the incident plane wave into vector spherical harmonics and writing the sphere's internal and external electromagnetic fields in terms of a vector harmonic expansion. Matching the boundary conditions at the sphere's surface allows the expansion coefficients of the incident, internal, and scattered fields to be determined as an infinite series of spherical multipole partial waves.

Analytical solution for the multiple sphere system

Following the notation in [8], extending the Lorenz-Mie theory to the multiple sphere system is more complex because the field external to a sphere i is a superposition of both the incident plane wave and the scattered fields from all the other spheres in the system:

$$\mathbf{E}_{ext} = \mathbf{E}_{inc} + \mathbf{E}_{sca} = \mathbf{E}_{inc} + \sum_{i=1}^{N_s} \mathbf{E}_{sca,i} \tag{1}$$

The incident and scattered wave for sphere i can then be written in terms of vector spherical wave functions centered about the origin of the ith sphere.

$$\mathbf{E}_{inc} = \sum_{n=1}^{L_i} \sum_{m=-n}^{n} \sum_{p=1}^{2} f^i_{mnp} \mathbf{N}^{(1)}_{mnp}(\mathbf{r} - \mathbf{r_i}) \tag{2}$$

$$\mathbf{E}_{sca,i} = \sum_{n=1}^{L_i} \sum_{m=-n}^{n} \sum_{p=1}^{2} a^i_{mnp} \mathbf{N}^{(3)}_{mnp}(\mathbf{r} - \mathbf{r_i}) \tag{3}$$

where $\mathbf{N}_{mnp}$ is the vector spherical wave function of type 1 (Bessel) or type 3 (Hankel) functions, n is the order, m is the degree, and p is the mode (1 for TM or 2 for TE). The incident field coefficient f^i_{mnp} depends on the field incident on sphere i, and the scattered field coefficients a^i_{mnp} are unknown coefficients that need to be solved for. To find analytical solutions for the scattered field coefficients, a truncation limit L_i for the infinite series of vector spherical wave functions can be determined to provide an acceptable level of convergence. In general, L_i will be proportional to the size parameter X_v. Applying the continuity equation at the surface of each sphere then leads to

a system of coupled linear equations for the scattered field coefficients for all the spheres in the systems:

$$a^i_{mnp} - \overline{a}^i_{np} \sum_{\substack{j=1,\\ j\neq i}}^{N_s} \sum_{n=1}^{L_i} \sum_{m=-n}^{n} \sum_{p=1}^{2} H^{i-j}_{mnpklq} a^j_{klq} = \overline{a}^i_{np} f^i_{mnp} \tag{4}$$

where $\overline{a}^i_{np}$ are the Mie coefficients of the sphere and H^{i-j} is the transformation matrix for outgoing spherical vector wave functions centered about origin j to an expansion about origin i. Equations 1-4 are the formal solution for the scattered field from all the spheres in the system. Equation 4 is a system of $2N_sL_i(L_i + 2)$ linear equations, assuming the same truncation limit L_i can be used for all the spheres in the system. Numerical methods are needed to iteratively solve this large system of linear equations.

To find the random orientation optical properties adds an additional level of complexity since the scattering must be averaged over all possible orientations and polarization states of the incident light. The T-Matrix approach [9, 10] is one method that is commonly used. Other computational approaches to calculate the optical properties of BC are discussed in a recent review [11].

Rayleigh-Debye-Gans Approximation

Rayleigh-Debye-Gans theory is a widely used approximation for light scattering from irregularly shaped particles [7, 12]. It relies on a length scaling argument, e.g. whether scattering from individual particles in a system will add constructively or destructively [7, 12]. It is valid in the range where particles are small relative to the wavelength of light:

$$2\pi a/\lambda \ll 1 \tag{5}$$

$$|m - 1| \ll 1 \tag{6}$$

The second condition, e.g. that particles be optically "soft", is not satisfied for BC [11].

S2 Machine Learning Approach

Graph Data Sets

Here we define a graph as $\mathcal{G} = (\mathcal{V}, \mathbf{A})$, where $\mathcal{V}$ is the vertex set of nodes $\{\nu_1, ..., \nu_n\}$ and $\mathbf{A} \in \mathbb{R}^{n\times n}$ is a symmetric adjacency matrix (as we consider only undirected graphs here). The adjacency matrix summarizes the connections, or edges, between the nodes. It can be either unweighted or weighted, such that elements of the adjacency matrix a_{ij} denote the edge weight between nodes ν_i and ν_j. For the unweighted case, $a_{ij} = 1$ if ν_i and ν_j are connected, $a_{ij} = 0$ otherwise. The graph's degree matrix $\mathbf{D}$ is a diagonal matrix of node degrees with elements $d_{ii} = \sum_j a_{ij}$ (non-diagonal elements are 0). The neighborhood $\mathcal{N}_k(i)$ are the nodes that are k steps away from the center node ν_i. We omit the subscript for neighborhood $k = 1$ steps away, e.g. the immediate neighborhood of the ith node is $\mathcal{N}(i)$.

Node features Each node ν_i has an associated feature vector $\mathbf{F}^{\nu_i}$. Here the features for each node are $\mathbf{F}^{\nu_i} = (X_v, Re(n_k), x_i, y_i, z_i)$, where (x_i, y_i, z_i) are the cartesian coordinates of the ith sphere. Since we make the assumption that aggregates are composed of isotropic, homogeneous spheres, X_v and $Re(n_k)$ could be considered "graph" features (e.g. $\mathbf{F}^u$) rather than "node" features, but in order to make the approach generalizable to systems with heterogenous spheres, we do not take this perspective here.

Edge features In general, edges e_{ij} can also have associated feature vectors $\mathbf{F}^{e_{ij}}$; in this case we consider only a single edge feature, which can be represented as the weights a_{ij} in the adjacency matrix. The edge feature we consider is the normalized distance between adjacent spheres, $r_{ij}/C = \sqrt{(x_i - x_j)^2 + (y_i - y_j)^2 + (z_i - z_j)^2}/C$, where C is the characteristic length scale (maximum distance between connected nodes) in the aggregate.

Graph Targets Here we focus on two supervised graph regression tasks, based on typical calculations needed for BC radiative forcing calculations and observational retrievals:

- prediction of the integral optical properties of BC (the efficiencies $\langle Q_{scat} \rangle$, $\langle Q_{abs} \rangle$, and $\langle Q_{ext} \rangle$ and the asymmetry parameter g)
- prediction of the angle-dependent elements of the scattering phase matrix $S_{ij}(\theta)$. We have mainly focused here on the prediction of $S_{11}(\theta)$, although we also tested the prediction of all $S_{ij}(\theta)$ elements for $j \geq i$ (Figure S16).

We denote the integral targets as Y_i^I and the angle dependent target $S_{11}(\theta)$ as Y_i^S. The predicted values for these targets from the GNN model are denoted as $\hat{Y_i^I}$ and $\hat{Y_i^S}$, respectively.

Graph Neural Network Models

The basic idea of graph neural networks for the graph regression task that we consider here is that each node, regardless of its number of neighbors, learns an embedding based upon the value of its feature vector $\mathbf{F}^{\nu_i}$ as well as the features of its neighbors $\mathbf{F}^{\nu_j}, j \in \mathcal{N}(i)$. Because the model is learned at the node level, the information from each of the nodes in the graph contributes to the fidelity of the model (e.g. the weights of the node-level model are learned based on all the nodes, and the neighborhoods of all the nodes, in the graphs). This node-level inductive bias also enables the zero-shot learning perspective since the model is able to immediately generalize to graphs with arbitrary numbers of nodes [2]. These node-level embeddings are then aggregated together and a graph level prediction is made. The model weights are learned by optimizing the graph-level predictions $\hat{Y_i^I}$ and $\hat{Y_i^S}$ against the true values Y_i^I and Y_i^S for the training data.

Optimization problem The model parameters $\mathbf{\Theta}$ are learned through back-propagation using the ADAM optimizer [13]. Here we assume mean-squared error (MSE) loss:

$$MSE = \frac{1}{n}\sum_{i=1}^{n}(\hat{Y_i^I} - Y_i^I)^2 + \frac{1}{m}\sum_{i=1}^{m}(\hat{Y_i^S} - Y_i^S)^2 \tag{7}$$

The learning rate is varied during training according to a 1 cycle learning rate policy with an upper learning rate boundary of $1e^{-4}$ [14]. We use L_2 regularization to minimize the model parameter weights with a weight decay parameter of $1e^{-8}$. We also investigated training the model on each set of targets individually (e.g. on only the first or only the second term on the right hand side of Eq. 7). We found that it was preferable to separately train the model to predict $\hat{Y^I}$ for the first task, while the prediction of $\hat{Y^S}$ was improved by training the model to predict both targets simultaneously.

Propagation rule The propagation rule is the method used to propagate information between nodes, capturing both the topological information about the graph structure and aggregating the node features. We tested several different approaches for the propagation module, including the simple graph convolution network (SGC) [15], the graph convolutional network (GCN) [16], and an interaction network (IN) [2, 17]. We denote each layer as $\mathbf{H}^l$, where $\mathbf{H}^l$ is a $\mathbb{R}^{n \times f_l}$ dimensional matrix, with n the number of nodes in the graph and f_l is the dimension of the node state, or embedding, at that layer (for the input layer, this is just the node feature vector $\mathbf{F}^{\nu_i}$).

- **SGC**: The Simple Graph Convolution (SGC) is described in [15]. The SGC differs from the other propagation rules that we tested, in that rather than having multiple layers, it reduces the complexity of multiple layers to a single linear transformation. The advantage of this approach is to remove potentially unnecessary complexity in GNN's and be more computationally efficient, with fewer fit parameters. The SGC is also more directly interpretable: the effect of the SGC is as a fixed filter on the graph spectral domain, effectively smoothing the local neighborhood so that nearby nodes have similar representations and predictions. Because there are no non-linear activation functions between layers, the effects of applying the SGC over multiple layers can be collapsed into a single linear transformation. The propagation rule to the Kth layer can be written as

$$\mathbf{H}^K = \mathbf{S}^K \mathbf{F}^{\nu_i} \mathbf{\Theta} \tag{8}$$

  where $\mathbf{S} = \tilde{\mathbf{D}}^{-\frac{1}{2}} \tilde{\mathbf{A}} \tilde{\mathbf{D}}^{-\frac{1}{2}}$ is the normalized adjacency matrix with added self-loops, and $\mathbf{\Theta}$ is a learned weight matrix.

- **GCN**: The Graph Convolutional Network (GCN) is described in [16]. The GCN's layer-wise propagation rule is based on a first-order approximation of spectral graph convolutions, which can be understood as localized spectral filters on graphs. This filter is localized to nodes that are at most 1 step away from the central node, e.g. the neighborhood $\mathcal{N}(i)$, and the propagation rule is

  $$\mathbf{H}^{l+1} = \sigma(\tilde{\mathbf{D}}^{-1/2}\tilde{\mathbf{A}}\tilde{\mathbf{D}}^{-1/2}\mathbf{H}^{l}\mathbf{\Theta}^{l}) \tag{9}$$

  where $\tilde{\mathbf{A}} = \mathbf{A} + \mathbf{I}_{N_s}$ is the adjacency matrix plus self-loops, $\tilde{\mathbf{D}}$ is the corresponding degree matrix, and $\mathbf{\Theta}^l$ is the layer-specific trainable-weight matrix. σ is a non-linear activation function; here we use the rectified linear unit (ReLU), which is defined as $\text{ReLU}(x) = max(0, x)$. Multiple convolutional layers can be stacked to create a deeper model, e.g. to propagate information to nodes that are K steps away. Each layer in this framework has a separate trainable-weight matrix.

- **WGCN**: The WGCN includes a weighted adjacency matrix, with distances scaled as r_{ij}/C, where C is the characteristic length scale (in this case the maximum possible distance between connected nodes).

- **IN**: The Interaction Network (IN) is described in [17]. The IN is based upon the idea of message-passing [18], where nodes "send" and "receive" messages along edges from their neighbors. (The GCN can also be reframed as message passing, as was done in [18]). In general, the IN can accommodate directed multi-graphs, although we consider only the case of an undirected graph with a single edge attribute between node i and node j, $\mathbf{F}^{e_{ij}}$. We also do not include the aggregation of all edge features in the global update, as in the full GraphNet block described in [2].

  Following the terminology in [2], the IN block consists of three "update" functions ϕ:

  $$\mathbf{F}'^{\mathbf{e_{ij}}} = \phi^{e}(\mathbf{F}^{\mathbf{e_{ij}}}, \mathbf{F}^{\nu_{\mathbf{i}}}, \mathbf{F}^{\nu_{\mathbf{j}}}) \tag{10}$$
  $$\mathbf{F}'^{\nu_{\mathbf{i}}} = \phi^{\nu_i}(\overline{\mathbf{F}}'^{\mathbf{e_{ij}}}, \mathbf{F}^{\nu_{\mathbf{i}}}) \tag{11}$$
  $$\mathbf{F}'^{\mathbf{u}} = \phi^{u}(\overline{\mathbf{F}}'^{\mathbf{e}}) \tag{12}$$

  and two "aggregation" functions ρ:

  $$\overline{\mathbf{F}}'^{\mathbf{e_{ij}}} = \rho^{e\to\nu}(E'_i) \tag{13}$$
  $$\overline{\mathbf{F}}'^{\nu} = \rho^{\nu\to u}(\mathcal{V}') \tag{14}$$

  where $E'_i = \{\mathbf{F}'^{\mathbf{e_{ij}}}\}$ is the set of all the message functions for the central node i and all of its neighbors j, and $\mathcal{V}' = \{\mathbf{F}'^{\nu_{\mathbf{i}}}\}_{i=1:N^{\nu}}$ is the set of all the nodes in the graph. The update functions ϕ^e, ϕ^{ν_i} and ϕ^u are denoted the edge-level, node-level, and graph-level models, respectively, and the primes indicate the updated state of the edges, node, and graph features. Each update function is a neural network with trainable weights. In this case both ϕ^e and ϕ^{ν_i} are implemented as MLP's with ReLU as a non-linear activation function between layers. As we are interested in graph-level prediction, the model is trained such that $\mathbf{F}'^{\mathbf{u}}$ predicts the graph targets.

  We try two variants, one where the center node's features are concatenated to the aggregated messages before passing to the node model (Eq. 11, denoted 'IN') and one where only the learned messages are passed to the node model $\mathbf{F}'^{\nu_{\mathbf{i}}} = \phi^{\nu}(\overline{\mathbf{F}}'^{\mathbf{e_{ij}}})$, denoted as 'IN2'. We use addition for the aggregation scheme over the edges for each node (Eq. 13). We test different aggregation functions for the global function (Eq. 14), as described in the next section.

Read-out operations for graph regression In order to aggregate the information from all of the nodes for the graph regression task, we use a graph pooling operation; graph pooling is required to be order-invariant (since a typical assumption for graph models is that nodes are permutation invariant– i.e. the identity of nodes should be interchangeable, as the graph-level prediction should be independent of the labeling of the nodes). We compare summation,

$$\mathbf{h}_G = \sum_{i=1}^{N} \mathbf{h}_i^L \tag{15}$$

averaging,

$$\mathbf{h}_G = \frac{1}{N}\sum_{i=1}^{N}\mathbf{h}_i^L \tag{16}$$

and max. pooling,

$$\mathbf{h}_G = max(\mathbf{h}_i^L) \tag{17}$$

as pooling operations, where $\mathbf{h}_G$ is the graph representation, and $\mathbf{h}_i^L$ is the representation of node ν_i at the final layer L.

After the graph pooling operation, we apply a final classifier, which is a fully connected layer. Here we use dropout (a regularization technique used to avoid overfitting where some of the elements of input tensor are randomly zeroed with a probability of p [19]) before the final classifier, with $p = 0.5$. The model is trained in an end-to-end fashion, where the weights for both the node level propagation/message passing layers and the final pooling and classification layer are optimized at once.

Impact of graph model on predictions

Propagation rules and graph layers Since the output of a GCN or IN layer is itself a graph G' (with the same connectivity as the original graph G), multiple layers can be stacked together. The layers correspond to the depth into a graph from each node that information is propagated, e.g. having k layers means that information can be propagated to neighbors that are at most k steps away. Because the information is effectively averaged over neighbors at each layer, however, having deep graph models is typically not as effective as having e.g. deep convolutional networks [20]. We tested both varying the number of layers before pooling and the number of hidden dimensions in each layer.

In Figure S5 we show a comparison of the MSE loss for the training data set as a function of the number of convolutional layers for the SGC vs. the GCN model on the task of predicting the integral optical properties $\hat{Y}_i$. The loss was significantly higher for the SGC, and did not improve with more layers, as the GCN model did, suggesting that the non-linearity of the GCN spectral filtering contributes to the improved prediction skill. The GCN model's performance significantly improved with more layers, suggesting that both propagating information further in the graph and allowing different weights to be learned at each layer improved the prediction relative to the SGC model. Similar results were seen for predicting $\hat{Y}^S$ or both $\hat{Y}^I$ and $\hat{Y}^S$, as in Eq. 7. Most of the improvement was seen in the addition of the first 4 layers (Figure S6); the validation loss did not significantly improve after 4 layers. The training loss for prediction of $\hat{Y}^S$ or both $\hat{Y}^I$ and $\hat{Y}^S$ similarly showed most improvement in the addition of the first 4 layers. The WGCN model performed slightly better than the GCN model with the same number of layers, indicating that including the distance between neighboring spheres as edge weights can improve the prediction of the model. The IN model's performance, even with a single graph layer, significantly out-performed the GCN variations; however, adding more than a single message passing layer for this model worsened, rather than improved, performance for the IN.

Impact of fully connected node Adding a special node to the graph that is connected to all the other nodes in the graph is one method commonly used to allow information to be propagated throughout the graph efficiently [18, 21], although it obscures the direct interpretation of the nodes in the graphs as the spheres of the aggregate, in this case. This approach is less computationally expensive than having e.g. a fully connected graph [18]. In this case we tested adding a fully connected node that has the same X_v and $Re(n_k)$ as the other spheres in the aggregate. To test models that include edge features, this node is assumed to be an averaged distance $\overline{r_{ij}}$ away from every other node in the graph (in this case we average only over nodes that are connected, e.g. only over the distances less than the characteristic length scale of the network).

Adding a fully connected node to the GCN (GCN-FC) improved the training loss slightly for the prediction of the integral optical properties, which suggests that allowing information to propagate further in the graph could improve prediction of Y_i. We denote this as "FC" in Figure S5. Adding a fully connected node improved performance on the validation sets for the IN model (Figure S10), but led to a bias in the performance on the zero-shot validation data set (Figure S11). For the prediction of the angle-dependent targets Y_s with the IN model, we found that the inclusion of the fully connected node led to slightly improved generalizability when comparing the predicted S_{11} integrated over the solid angle to the values of g (Eq. 4 in the main manuscript) compared to no fully connected node for the zero-shot validation data set.

Impact of Node Model and Message Size For the IN model, we investigated different approaches for the node and edge level models. We varied the number of layers in both the node and edge level models (as discussed above, they are each MLP's with ReLU as a non-linear activation function between layers), as well as the size of the learned message. In Figure S7, we show that inputting both the concatenated messages from all the neighbors as well as the central node's features ('IN') performed better than passing only the learned messages to the node model ('IN2'). Having several layers in the node and edge models decreased MSE loss for the training and validation data sets compared with only a single layer ($H^e_{L=3}$,$H^v_{L=3}$ vs. $H^e_{L=1}$,$H^v_{L=1}$). This suggests that more complex approaches for the node and edge models, such as using spherical harmonic filters [22], could further improve predictions by more fully exploiting the connection to the Mie theory solution for each individual sphere as influenced by the incident light and all the other spheres in the system (Eq. 1). We also found that, while increasing the size of the learned message slightly improved the MSE loss for the training and validation sets for both predicting $\hat{Y}^S$ and predicting $\hat{Y}^I$ (Figure S8), restricting the size to 100 improved the zero-shot generalization performance on the zero-shot validation data set (Figure S9). This bottle-necking was previously observed to provide improved interpretability of the learned messages in an IN applied to an n-body physics simulation [23].

Impact of readout function The readout operations of average and max pooling performed better for the training and validation sets for both the GCN and IN for the prediction of $\hat{Y}^I$ (Figure S10). Summation gave a lower MSE loss than the mean global pooling for the training and validation data sets for the prediction of Y^S; however the generalization performance was typically better for the mean and max. pooling cases (Figure S12). Intuitively we might expect summation to provide the best performance, as the total optical properties of the aggregate are due to the contributions of all the individual spheres; however because scattering and absorption can add constructively and destructively, this suggests the mean is more informative.

Impact of characteristic length scale As discussed in the main text, we generate graphs for each BC fractal aggregate from their dimensionless cartesian coordinates by connecting spheres with center positions closer than the characteristic length scale C of the network. Since this length scale will impact the resulting graph structure and potentially impact the performance of the graph model, we also tested whether using a different characteristic length scale would impact the predictions. We tried characteristic length scales of $C = X_v Log(N_s)$ and $C = X_v Log(N_s)/Log(Log(N_s))$. We found that the training and validation loss were both lower for $C = X_v Log(N_s)$ for all the prediction tasks. We also found that the model demonstrated superior zero-shot performance for $C = X_v Log(N_s)$.

One might also consider using a fully connected graph (since in general all spheres interact with one another), but this becomes prohibitively expensive for large N_s, as the number of edges would grow as N_s^2 rather than $Log(N_s)$ (Figure 3e in the main manuscript). We note a similar assumption was made for CELES, the CUDA-accelerated version of MSTM, which sub-divided neighboring spheres into subsets, to block-diagnolize portions of the T-matrix in order to parallelize calculations and improve computational performance [24].

Impact of training sample size, batch size, and maximum graph size We vary the number of graphs s used in the training data set. Figure S13 shows the training loss and validation loss for the GCN and IN as a function of the number of samples in the training data set. The model is fairly efficient in terms of its usage of the data, and the most significant improvement is seen when increasing the sample size up to $s \sim 10000$ samples, with less significant improvement in the training loss for the prediction of $\hat{Y}^I$ with larger sample sizes. The loss for predicting $\hat{Y}^S$ showed a slightly more linear decrease with increasing s for the IN, but the majority of the improvement was also seen for increasing the number of samples up to $s \sim 15000$.

For zero-shot learning we also investigated how varying the maximum number of nodes $N_{s,max}$ allowed in graphs in the training data sets impacted the generalizability of the model to larger graphs (Figure S14). Smaller $N_{s,max}$ significantly increased the bias for the zero-shot prediction, although the model still showed some skill in zero-shot prediction for $N_{s,max} \geq 40$.

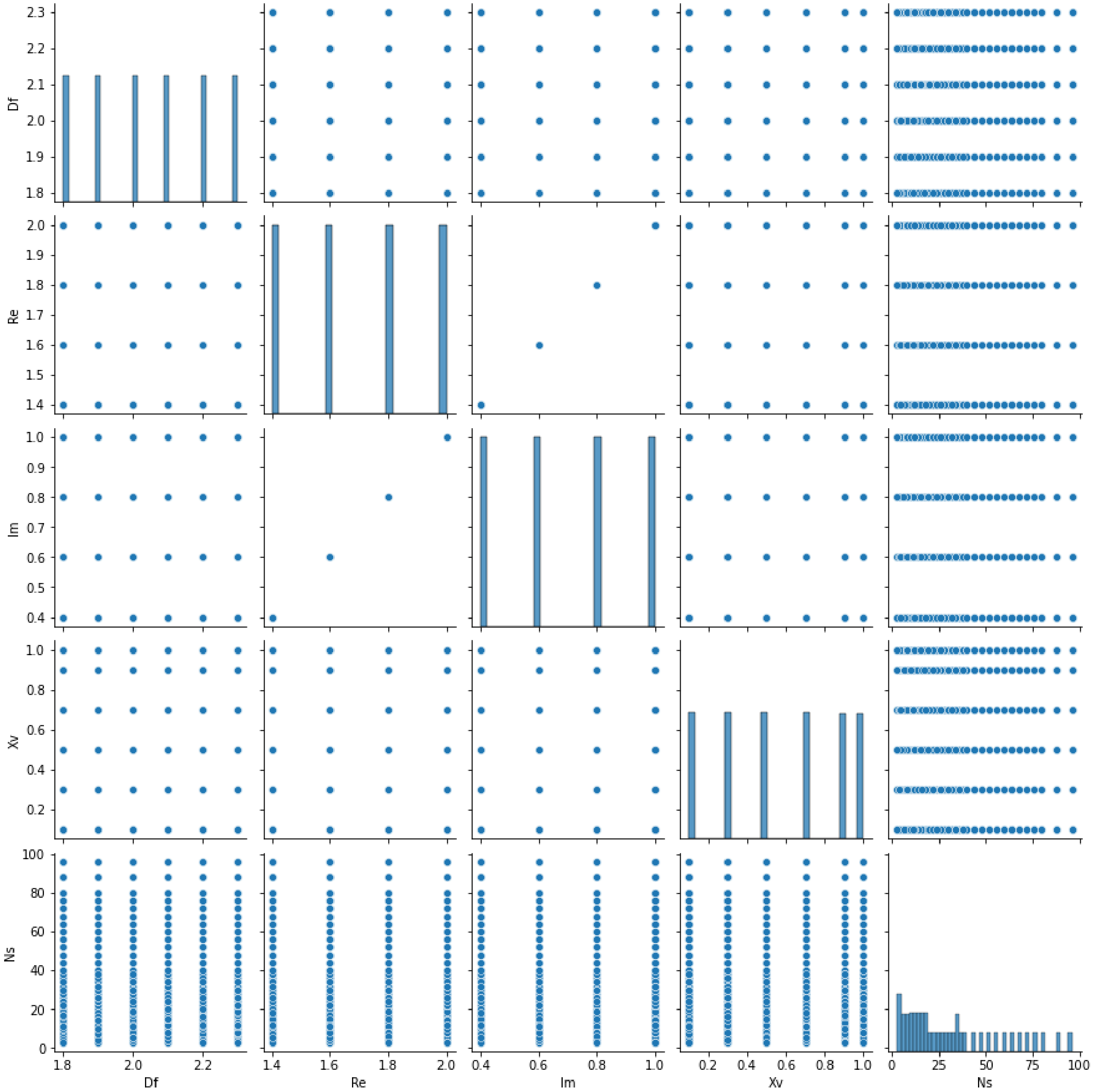

Figure S1: Distribution of parameters in training, validation, and test data sets ($N_s < 100$).

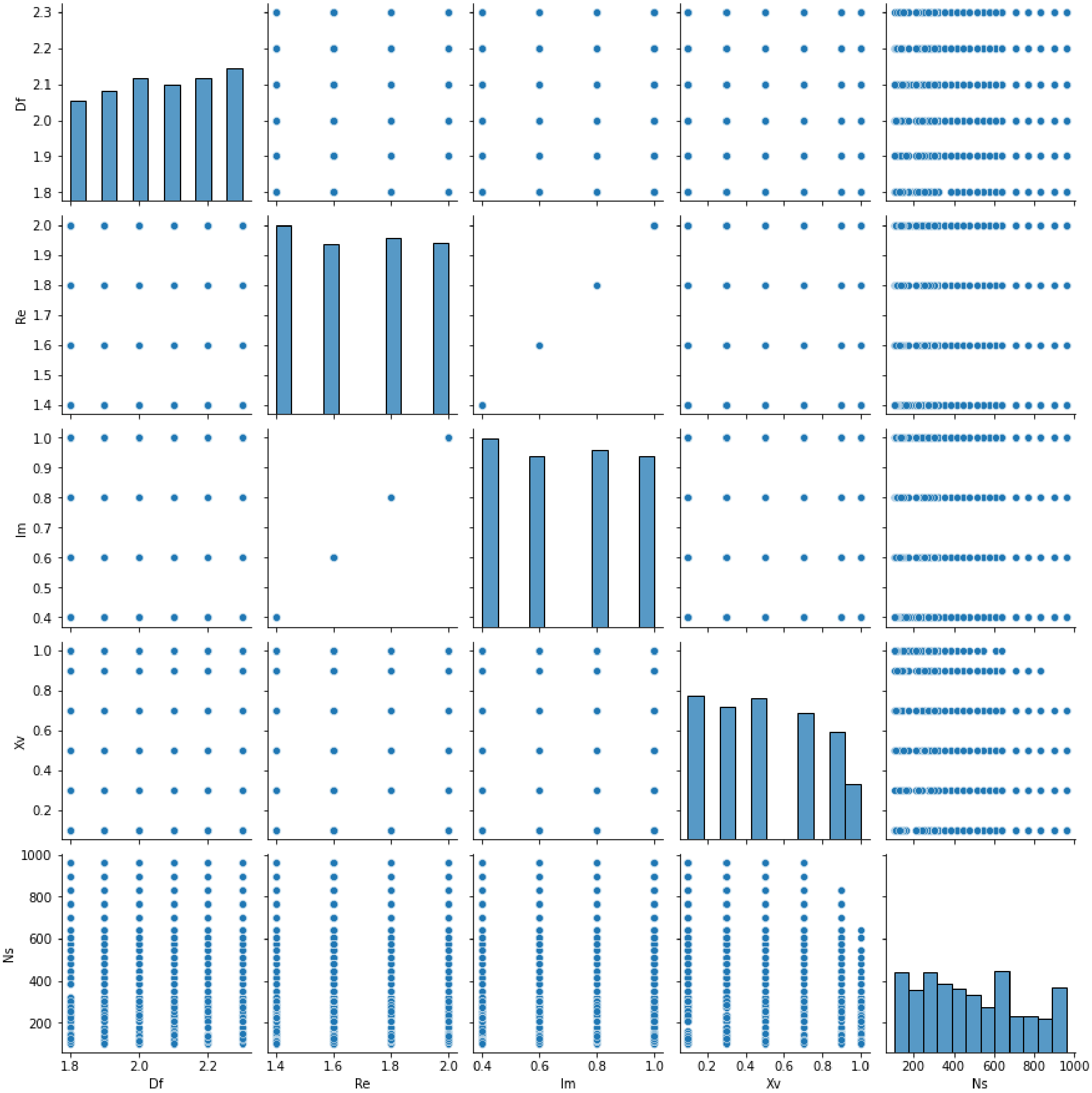

Figure S2: Distribution of parameters in zero shot test and validation data sets ($N_s > 100$).

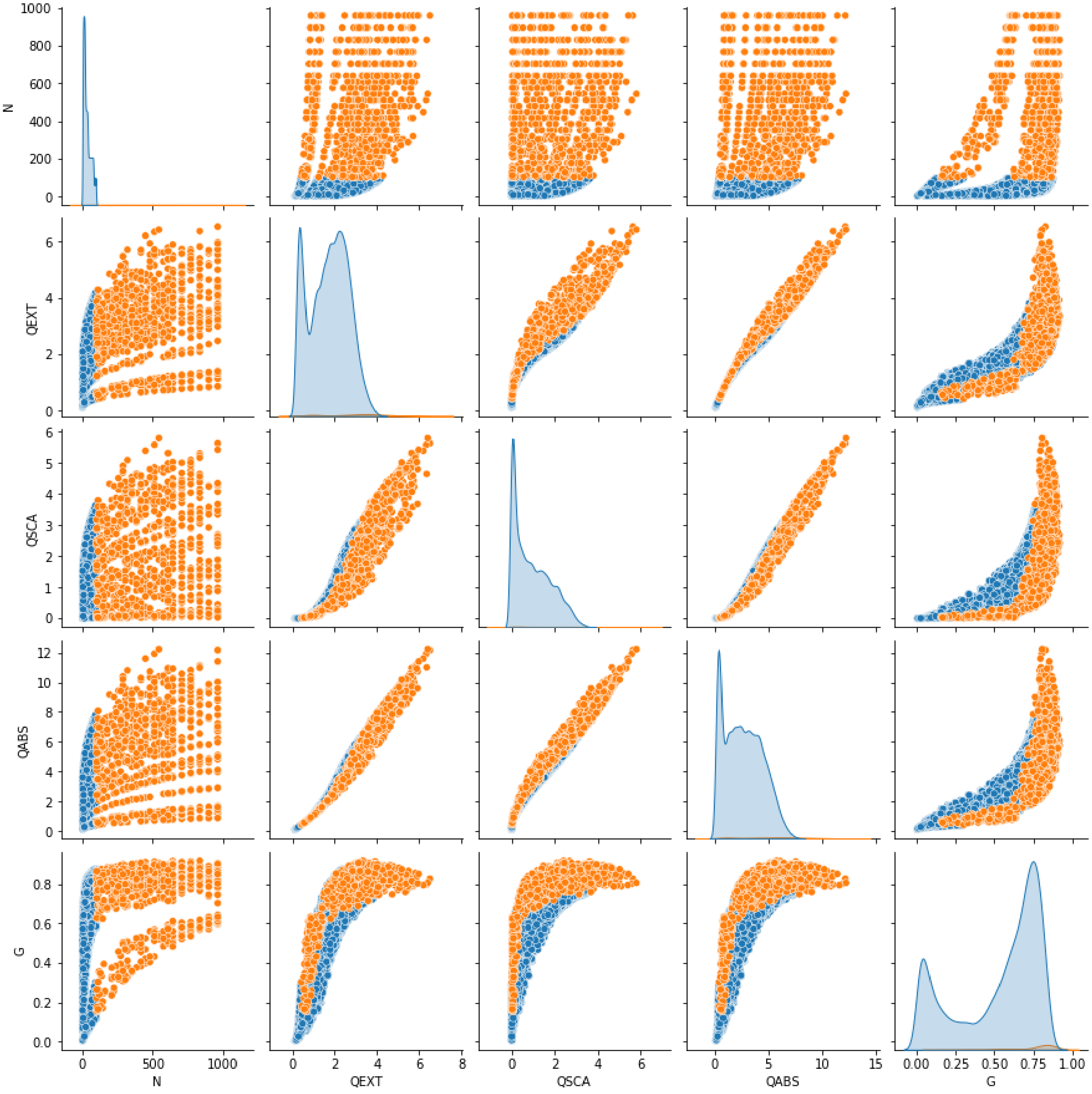

Figure S3: Integral optical properties as a function of the parameters for the large ($N_s > 100$, orange) and small ($N_s < 100$, blue) aggregates.

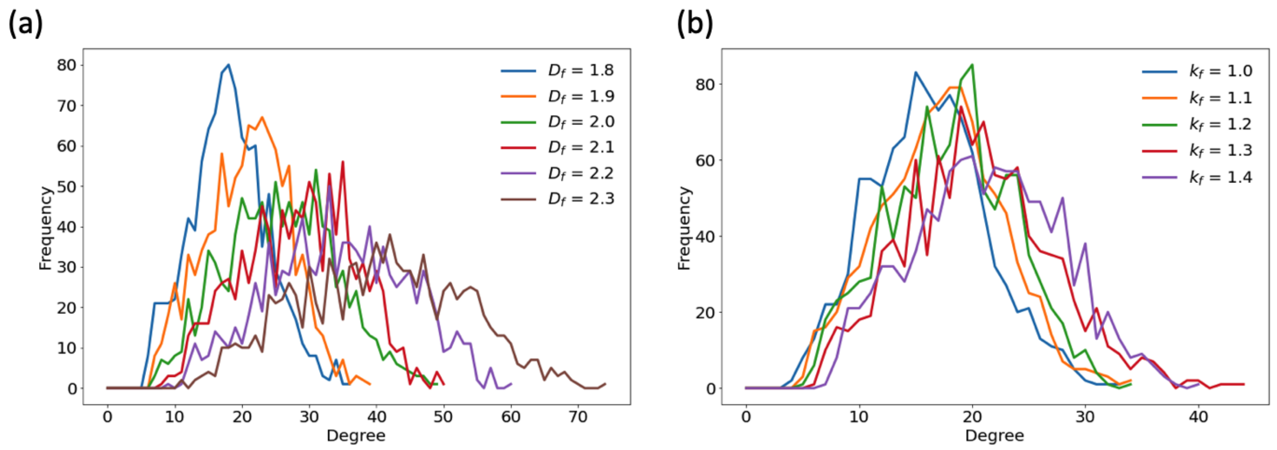

Figure S4: **(a.)** The degree distribution for aggregates with $N_s = 1024$ spheres, $k_f = 1.2$, and D_f as given in the legend. **(b.)** Degree distributions for an aggregate with $N_s = 1024$ spheres, $D_f = 1.8$, and k_f as given in the legend.

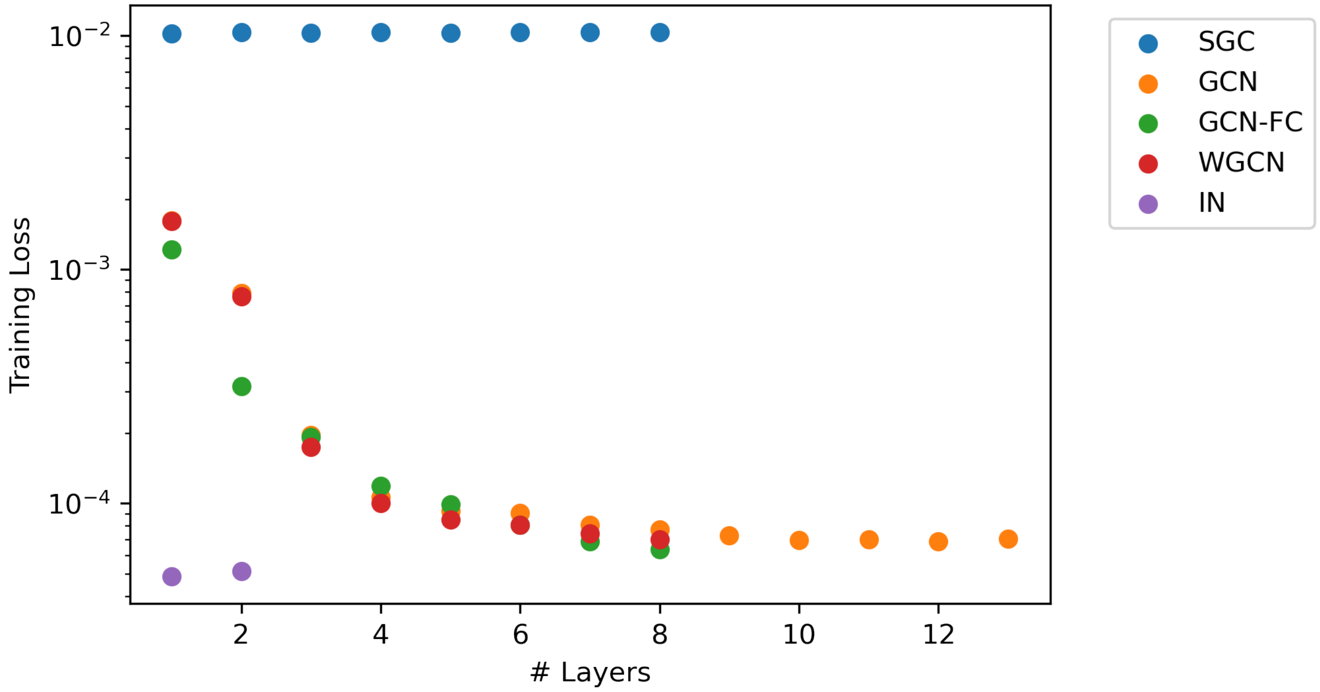

Figure S5: Training loss for the prediction of integral optical properties for the models with different number of layers.

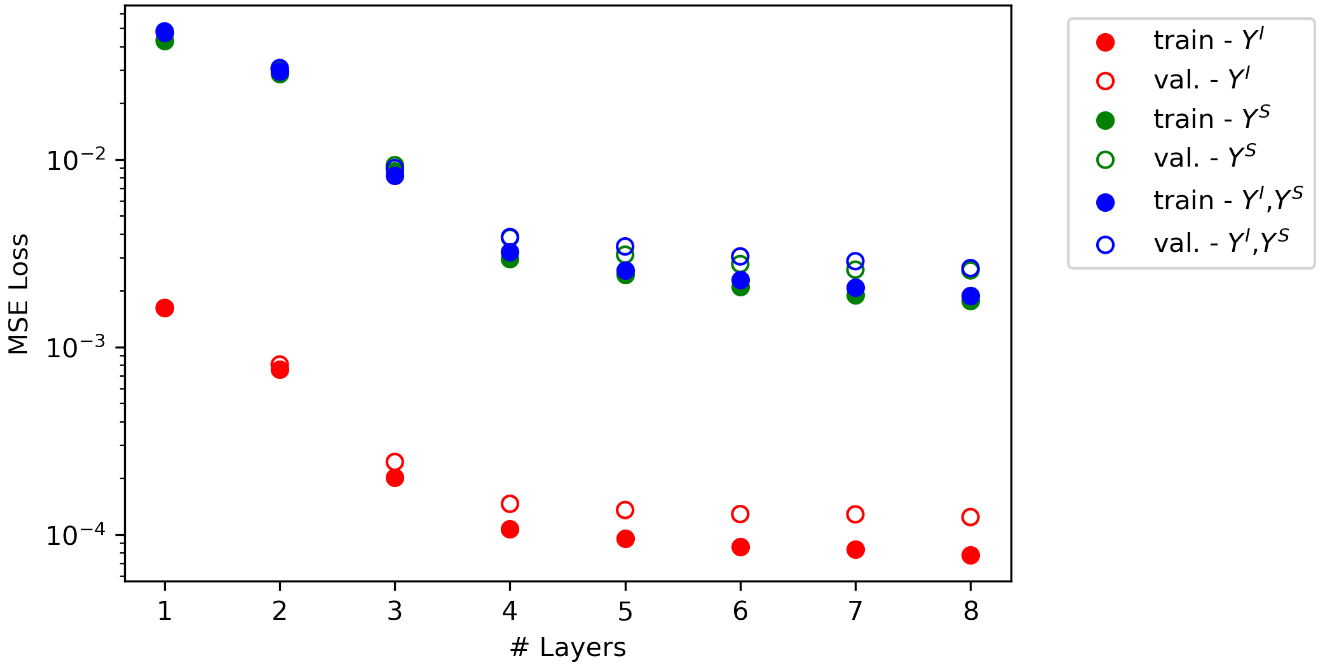

Figure S6: Training and validation loss for the prediction of task of different targets (Y^I, Y^S or Y^I and Y^S) for the GCN model with different number of layers.

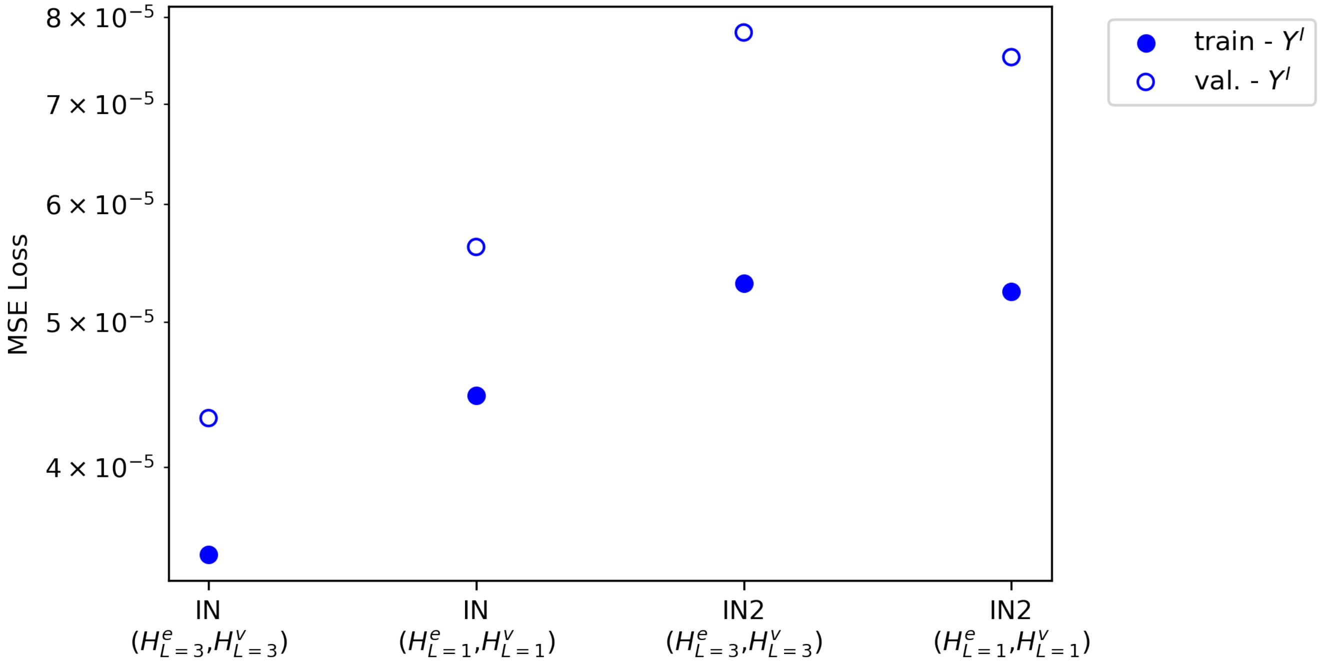

Figure S7: Training and validation loss for the prediction of Y^I for different IN model architectures. 'IN' concatenates the central node features with the aggregated edges, as given in Eq. 11, while 'IN2' does not include the central node features. H^e_L and H^v_L indicate the number of layers in the MLP's for the edge and node models, respectively.

Table S1: Parameters of BC aggregates for training, validation, and test data sets.

Data set	Training/Val./Test Sets	Zero-shot Val./Test Sets
N_s	8-96	104 - 960
D_f	1.8, 1.9, 2.0, 2.1, 2.2, 2.3	
k_f	1.2	
n_k	$1.4+0.4i$, $1.6+0.6i$, $1.8+0.8i$, $2.0+1.0i$	
X_v	0.1, 0.3, 0.5, 0.7, 0.9, 1.0	
Total #	57,556	880

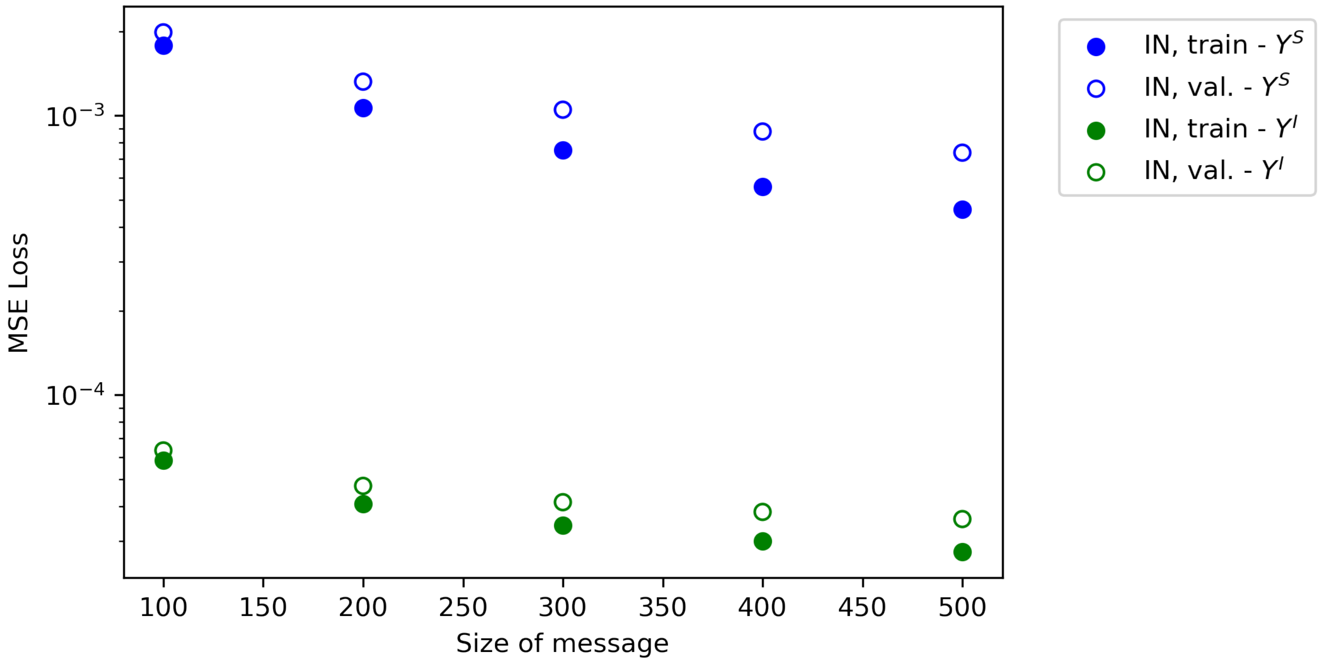

Figure S8: Training and validation loss for the prediction of different targets (Y^I or Y^S) for the IN model with different sizes for the learned message.

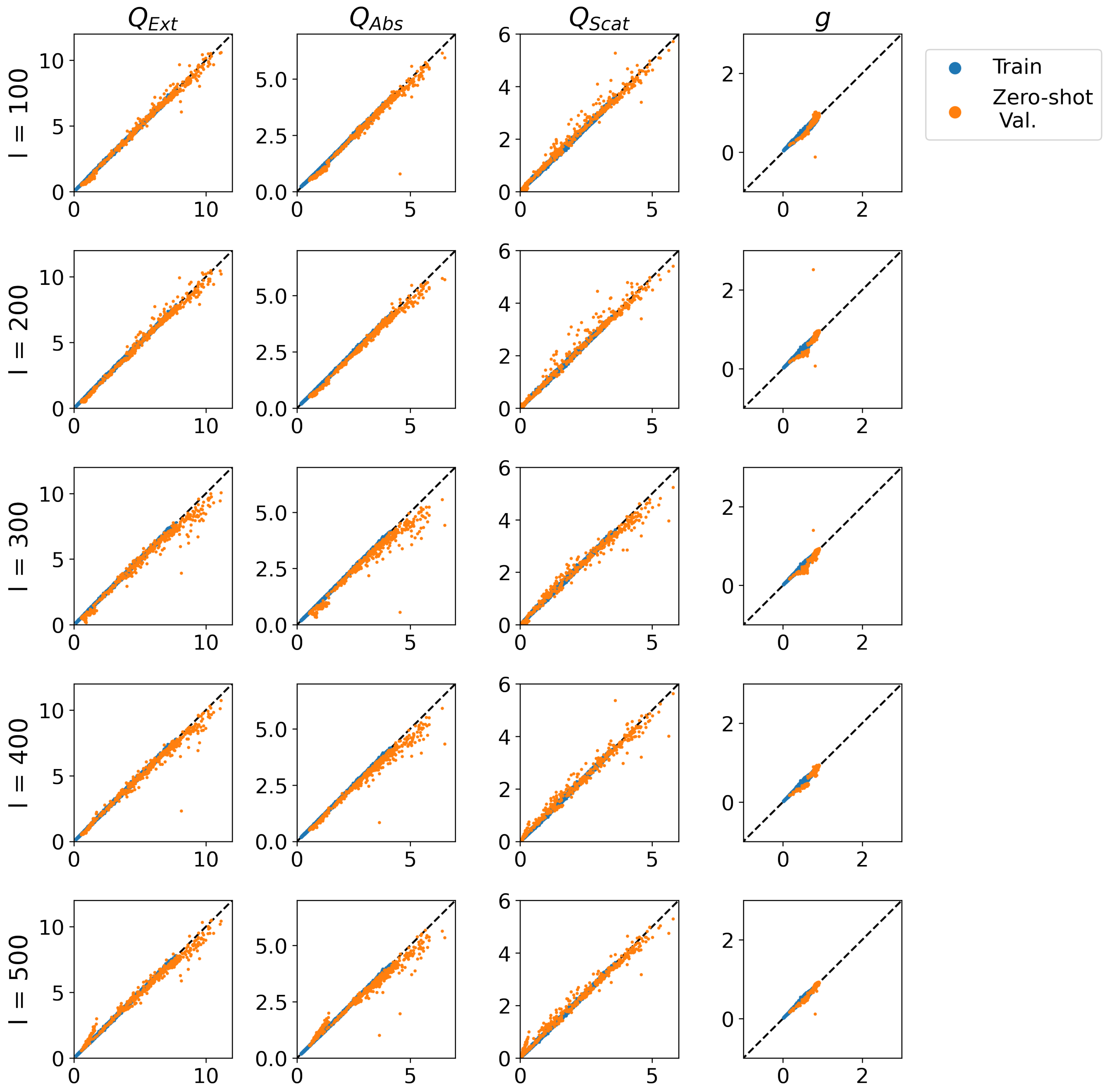

Figure S9: Predictions for the integral optical properties for the training and zero-shot validation data sets using an IN model. Each row shows the true vs. predicted values for the model as trained with different message sizes.

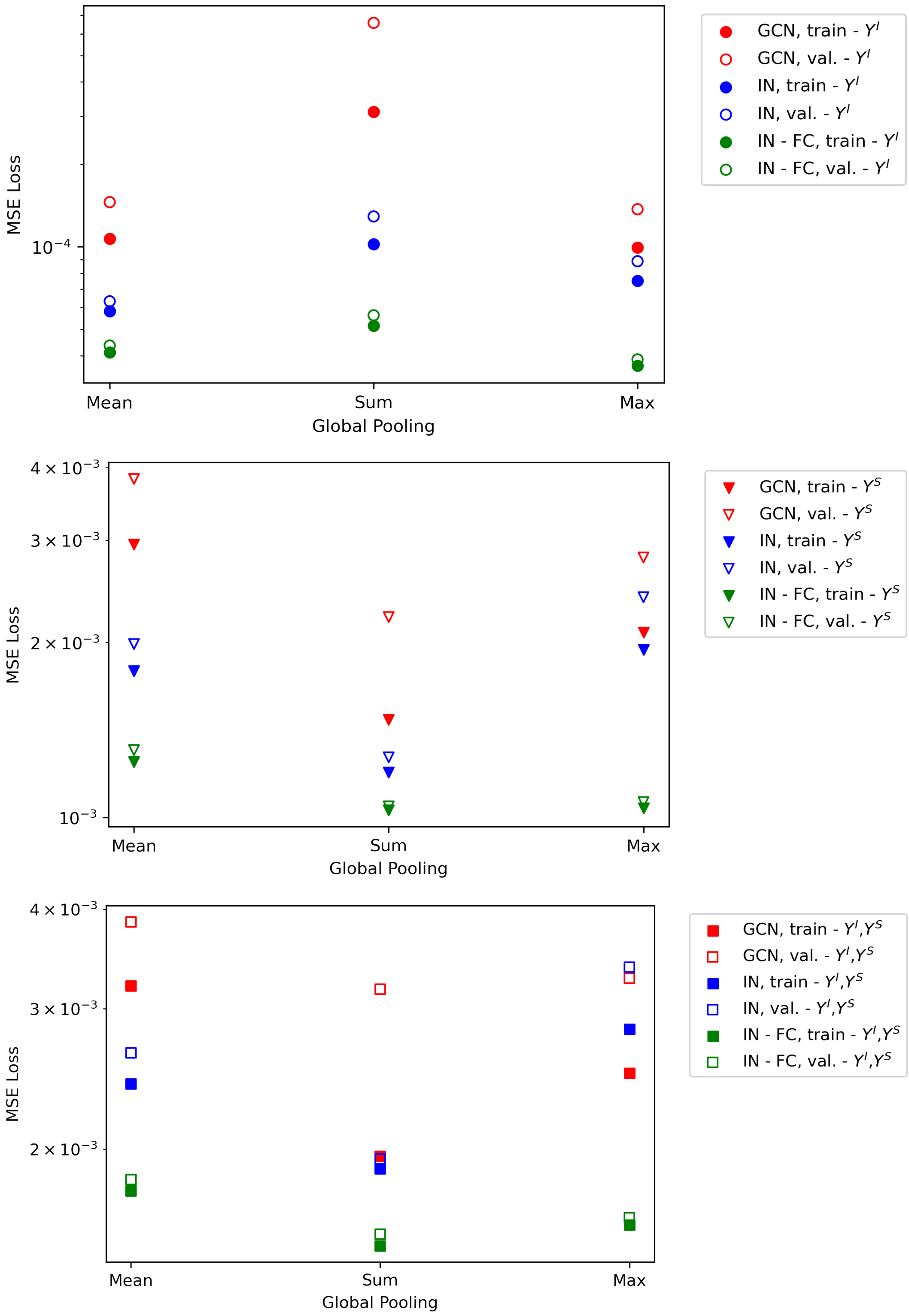

Figure S10: Training and validation loss for the prediction of the GCN model and the IN model with different graph readout functions and targets. The GCN model has 4 layers, a hidden dimension size of 400, and dropout of 0.5, using a global mean pooling layer. The IN model has a message size of 100.

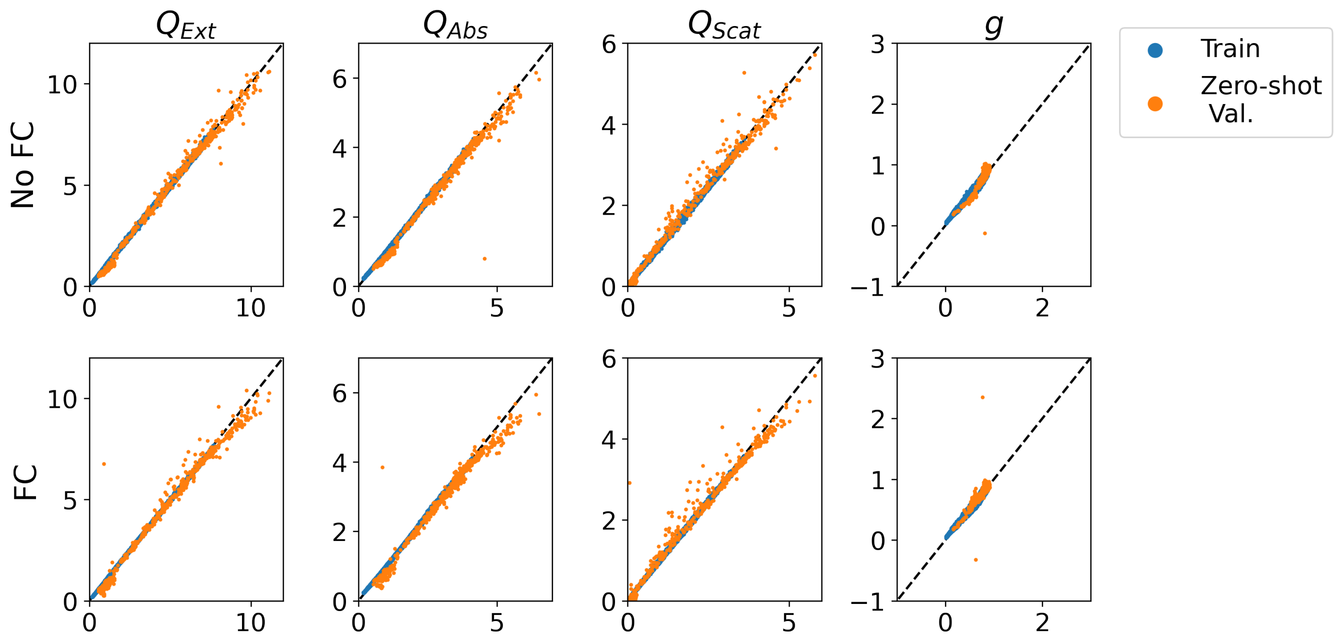

Figure S11: Predictions for the integral optical properties for the training and zero-shot validation data sets using an IN model with a message size of 100. Each row shows the true vs. predicted values for the model as trained without a fully connected node (No FC) and with a fully connected node (FC) in each graph.

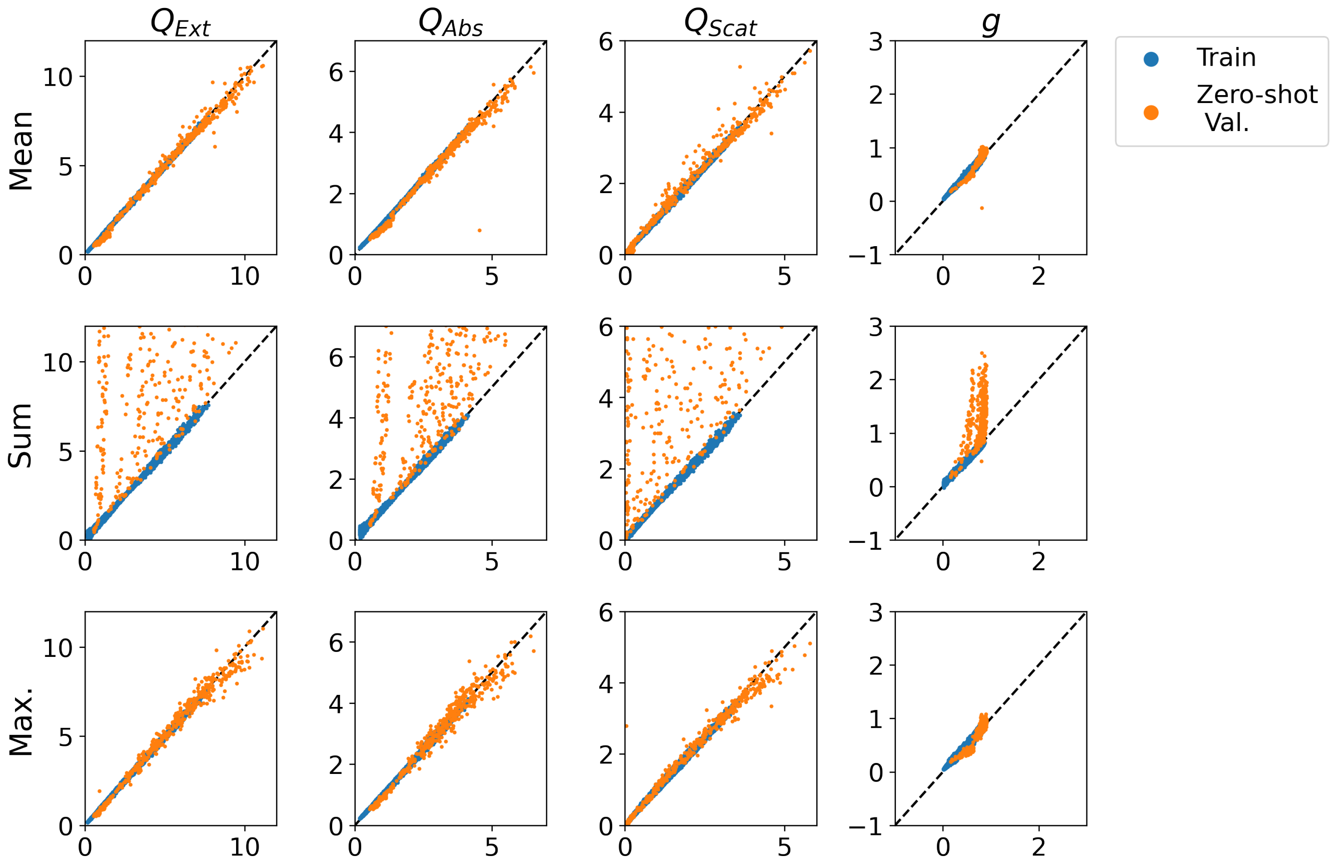

Figure S12: Predictions for the integral optical properties for the training and zero-shot validation data sets using an IN model with a message size of 100. Each row shows the true vs. predicted values for the model as trained with different global pooling functions.

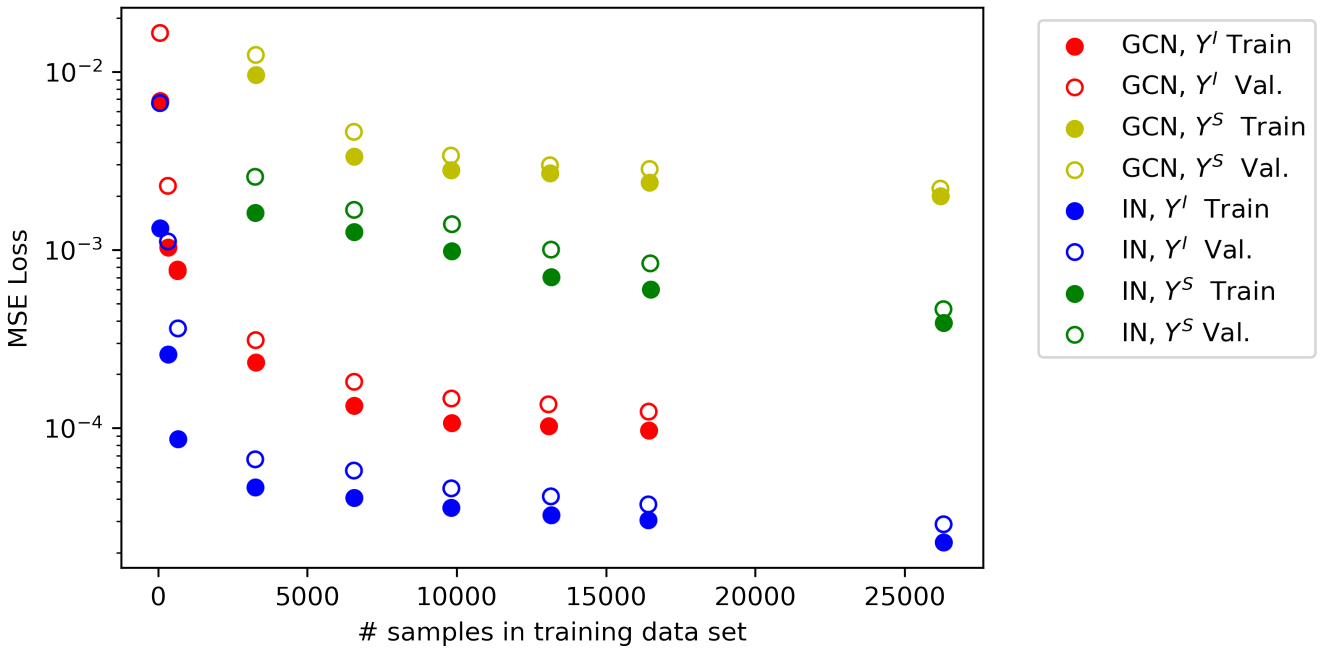

Figure S13: Training and validation loss for the prediction of integral optical properties for the GCN model and the IN model with different training sample size and targets. The GCN model has 4 layers, a hidden dimension size of 400, and dropout of 0.5, using a global mean pooling layer.

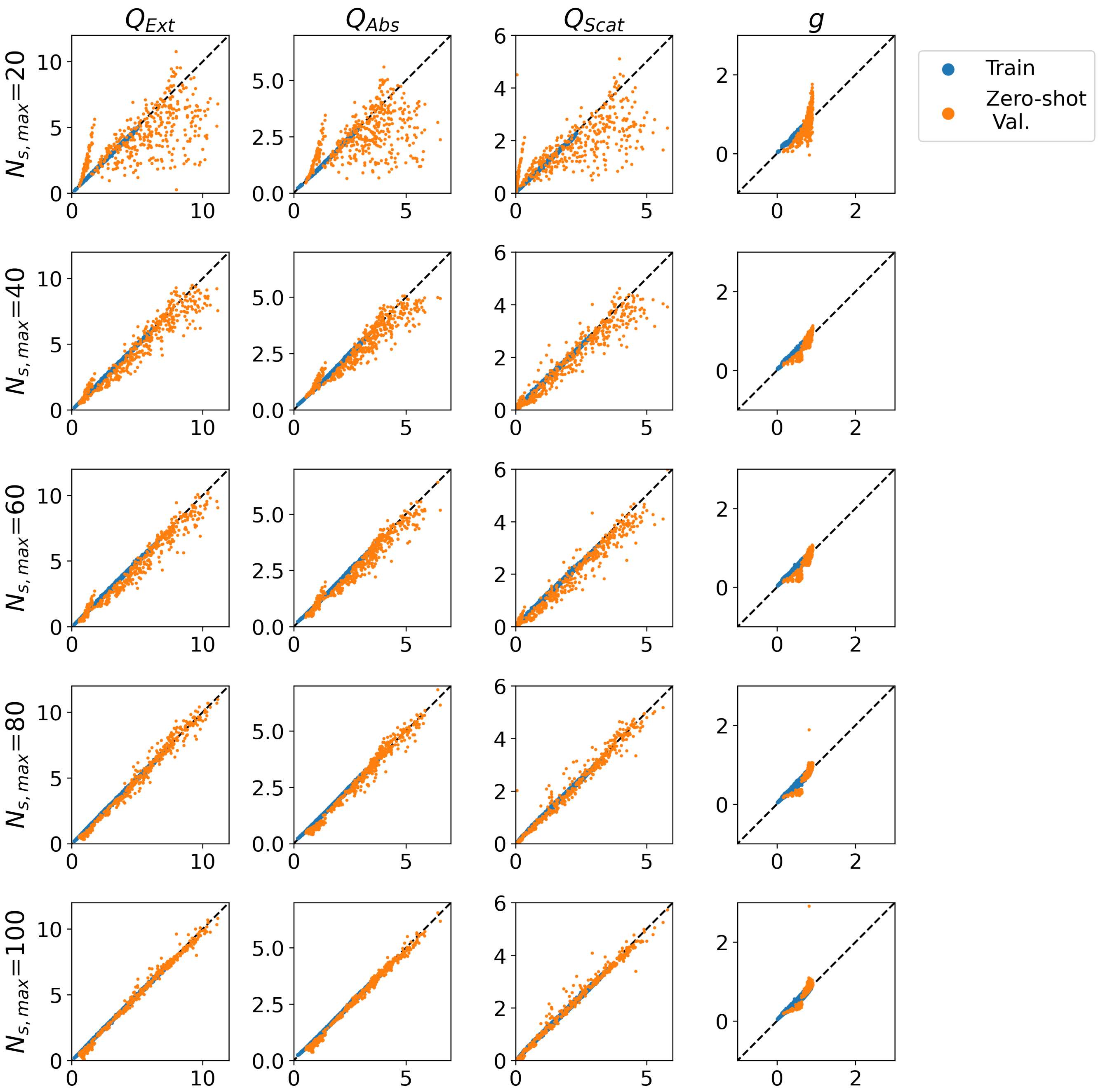

Figure S14: Predictions for the integral optical properties for the training and zero-shot validation data sets using an IN model with a message size of 100. Each row shows the true vs. predicted values for the model as trained with different maximum aggregate sizes in the training data sets.

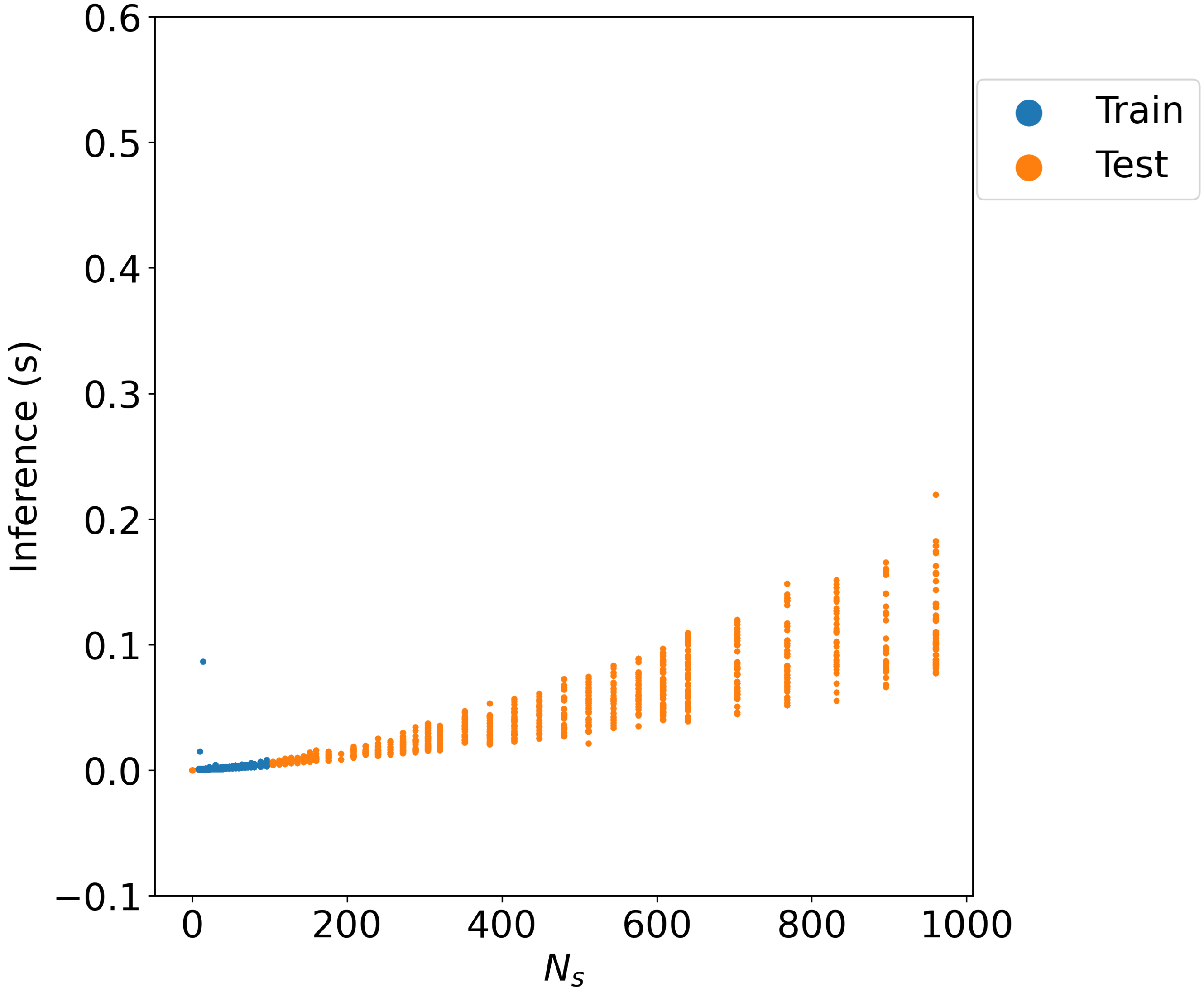

Figure S15: Inference time in seconds as a function of the size of the aggregate for the trained IN model for predictions on the training data set and zero-shot test data sets. The inference time here was estimated on a CPU using the trained model weights.

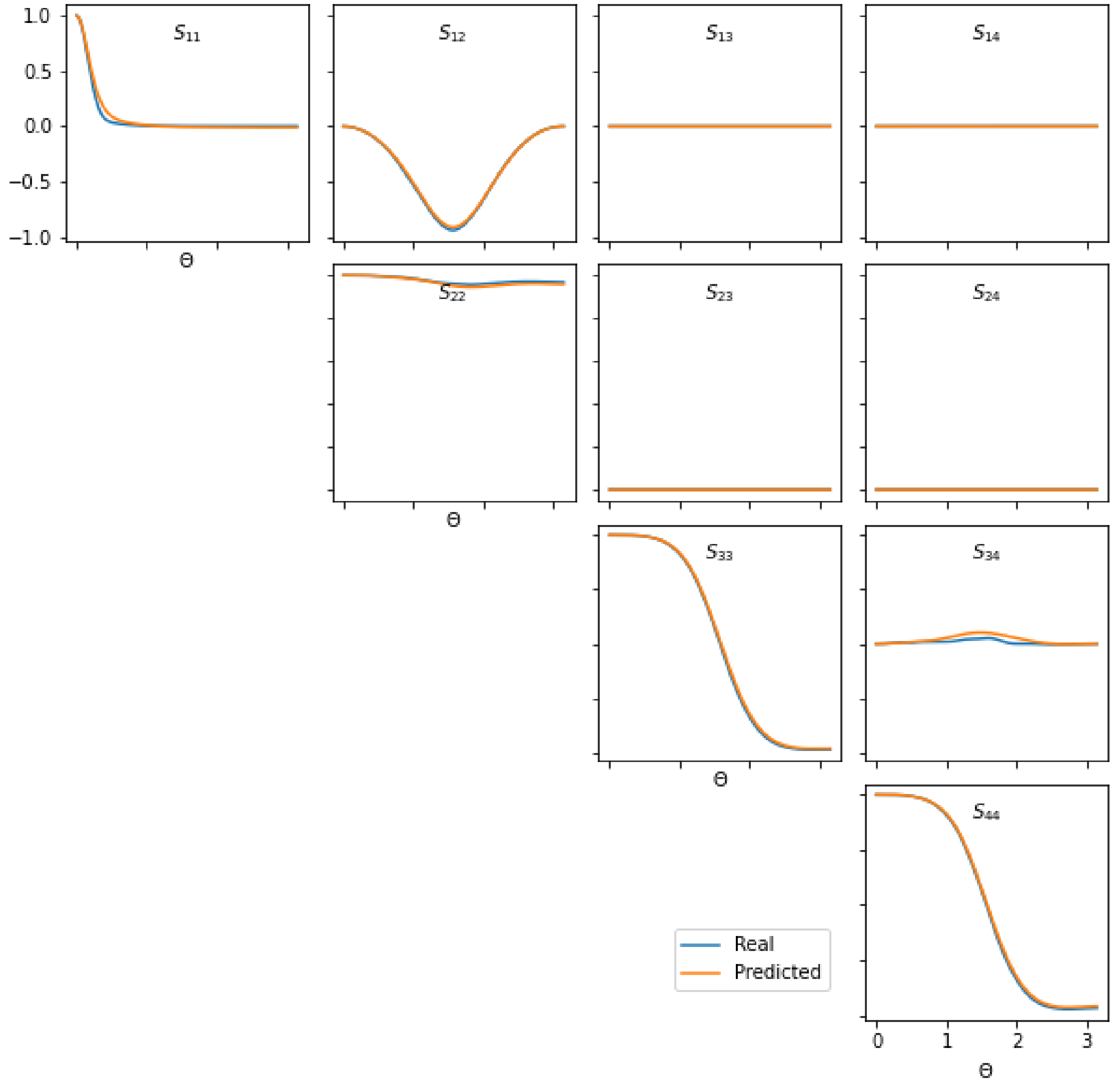

Figure S16: Predictions for S_{ij} for an aggregate in the test data set with $N_s = 960$, $X_v = 0.5$, and $n_k = 1.4 + 0.4i$.

References

[1] M. M. Bronstein, J. Bruna, Y. LeCun, A. Szlam, and P. Vandergheynst, "Geometric deep learning: going beyond euclidean data," *IEEE Signal Processing Magazine*, vol. 34, no. 4, pp. 18–42, 2017.

[2] P. W. Battaglia, J. B. Hamrick, V. Bapst, A. Sanchez-Gonzalez, V. Zambaldi, M. Malinowski, A. Tacchetti, D. Raposo, A. Santoro, R. Faulkner, *et al.*, "Relational inductive biases, deep learning, and graph networks," *arXiv preprint arXiv:1806.01261*, 2018.

[3] J. Zhou, G. Cui, Z. Zhang, C. Yang, Z. Liu, L. Wang, C. Li, and M. Sun, "Graph neural networks: A review of methods and applications," *arXiv preprint arXiv:1812.08434*, 2018.

[4] Z. Wu, S. Pan, F. Chen, G. Long, C. Zhang, and S. Y. Philip, "A comprehensive survey on graph neural networks," *IEEE transactions on neural networks and learning systems*, 2020.

[5] Z. Zhang, P. Cui, and W. Zhu, "Deep learning on graphs: A survey," *IEEE Transactions on Knowledge and Data Engineering*, 2020.

[6] G. Mie, "Beiträge zur optik trüber medien, speziell kolloidaler metallösungen," *Annalen der physik*, vol. 330, no. 3, pp. 377–445, 1908.

[7] C. F. Bohren and D. R. Huffman, *Absorption and scattering of light by small particles.* John Wiley & Sons, 2008.

[8] D. W. Mackowski and M. I. Mishchenko, "A multiple sphere t-matrix fortran code for use on parallel computer clusters," *J. Quant. Spectrosc. Radiat. Transfer*, vol. 112, pp. 2182–2192, 2011.

[9] D. W. Mackowski, "Calculation of total cross sections of multiple-sphere clusters," *JOSA A*, vol. 11, no. 11, pp. 2851–2861, 1994.

[10] D. W. Mackowski and M. I. Mishchenko, "Calculation of the t matrix and the scattering matrix for ensembles of spheres," *JOSA A*, vol. 13, no. 11, pp. 2266–2278, 1996.

[11] M. Kahnert and F. Kanngießer, "Modelling optical properties of atmospheric black carbon aerosols," *Journal of Quantitative Spectroscopy and Radiative Transfer*, vol. 244, p. 106849, 2020.

[12] C. Sorensen, "Light scattering by fractal aggregates: a review," *Aerosol Science & Technology*, vol. 35, no. 2, pp. 648–687, 2001.

[13] D. P. Kingma and J. Ba, "Adam: A method for stochastic optimization," *arXiv preprint arXiv:1412.6980*, 2014.

[14] L. N. Smith and N. Topin, "Super-convergence: Very fast training of neural networks using large learning rates," in *Artificial Intelligence and Machine Learning for Multi-Domain Operations Applications*, vol. 11006, p. 1100612, International Society for Optics and Photonics, 2019.

[15] F. Wu, A. Souza, T. Zhang, C. Fifty, T. Yu, and K. Weinberger, "Simplifying graph convolutional networks," in *International conference on machine learning*, pp. 6861–6871, PMLR, 2019.

[16] T. N. Kipf and M. Welling, "Semi-supervised classification with graph convolutional networks," *arXiv preprint arXiv:1609.02907*, 2016.

[17] P. W. Battaglia, R. Pascanu, M. Lai, D. Rezende, and K. Kavukcuoglu, "Interaction networks for learning about objects, relations and physics," *arXiv preprint arXiv:1612.00222*, 2016.

[18] J. Gilmer, S. S. Schoenholz, P. F. Riley, O. Vinyals, and G. E. Dahl, "Neural message passing for quantum chemistry," in *International Conference on Machine Learning*, pp. 1263–1272, PMLR, 2017.

[19] N. Srivastava, G. Hinton, A. Krizhevsky, I. Sutskever, and R. Salakhutdinov, "Dropout: a simple way to prevent neural networks from overfitting," *The journal of machine learning research*, vol. 15, no. 1, pp. 1929–1958, 2014.

[20] Q. Li, Z. Han, and X.-M. Wu, "Deeper insights into graph convolutional networks for semi-supervised learning," in *Proceedings of the AAAI Conference on Artificial Intelligence*, vol. 32, 2018.

[21] F. Scarselli, M. Gori, A. C. Tsoi, M. Hagenbuchner, and G. Monfardini, "The graph neural network model," *IEEE transactions on neural networks*, vol. 20, no. 1, pp. 61–80, 2008.

[22] J. Klicpera, J. Groß, and S. Günnemann, "Directional message passing for molecular graphs," *arXiv preprint arXiv:2003.03123*, 2020.

[23] M. Cranmer, A. Sanchez-Gonzalez, P. Battaglia, R. Xu, K. Cranmer, D. Spergel, and S. Ho, "Discovering symbolic models from deep learning with inductive biases," *arXiv preprint arXiv:2006.11287*, 2020.

[24] A. Egel, L. Pattelli, G. Mazzamuto, D. S. Wiersma, and U. Lemmer, "Celes: Cuda-accelerated simulation of electromagnetic scattering by large ensembles of spheres," *Journal of Quantitative Spectroscopy and Radiative Transfer*, vol. 199, pp. 103–110, 2017.
